# Stat3 Is Required to Maintain the Full Differentiation Potential of Mammary Stem Cells and the Proliferative Potential of Mammary Luminal Progenitors

**DOI:** 10.1371/journal.pone.0052608

**Published:** 2012-12-20

**Authors:** Anna D. Staniszewska, Sara Pensa, Maria M. Caffarel, Lisa H. Anderson, Valeria Poli, Christine J. Watson

**Affiliations:** 1 Department of Pathology, University of Cambridge, Cambridge, United Kingdom; 2 Molecular Biotechnology Center, Department of Genetics, Biology and Biochemistry, University of Turin, Turin, Italy; Baylor College of Medicine, United States of America

## Abstract

Stat3 has a defined role in mammary gland where it is a critical mediator of cell death during post-lactational regression. On the other hand, Stat3 is required for the self-renewal of embryonic stem cells and is sufficient for the induction of a naïve pluripotent state in epiblast stem cells. Mammary stem cells (MaSCs) have a high capacity for self-renewal and can grow robustly in transplantation experiments *in vivo*. However, a role for Stat3 in MaSCs has not been investigated. Here we show that depletion of Stat3 from basal cells results in reduced primary transplantation efficiency and diminishes the potential to generate ductal, but not alveolar, outgrowths. In addition, Stat3 is required for maximal proliferation of luminal progenitors.

## Introduction

The transcription factor Signal Transducer and Activator of Transcription (Stat) 3 is constitutively expressed in a wide variety of tissues. Stat3 is activated by various cytokines and growth factors such as OSM, LIF, IL-6, IL-10, IL-17, IL-23, leptin, EGF, and interferons, as well as the proto-oncogenes and oncogenes c-Src, c-Abl, Met, and ErbB2 [1]. Leukaemia inhibitory factor (LIF), which belongs to the IL-6 family of cytokines, is indispensable for self-renewal of mouse embryonic stem cells (mESCs) and maintenance of their undifferentiated state [2]. LIF, after binding to the LIFR/gp130 heterodimer, can trigger three signalling pathways, namely JAK/Stat3, PI3K/Akt and SHP2/MAPK. However, the Stat3 branch of LIF signalling has been demonstrated to play a central role in the regulation of self-renewal and pluripotency of mESCs [3]. In fact, overexpression of a dominant negative variant of Stat3 in mESCs leads to loss of pluripotency and enhanced cell differentiation [4]. Furthermore, Stat3 activation is sufficient to maintain the undifferentiated state of mESCs, as demonstrated in a study using a fusion protein between Stat3 and the ligand binding domain of the estrogen receptor (ER) [5]. Additionally, deletion of *Stat3* causes embryonic lethality as *Stat3* ^−/−^ embryos have reduced growth of the inner cell mass (ICM) and show rapid degeneration between days E6.5– E7.5 [6]. Thus, Cre-loxP systems have been used to investigate the role of Stat3 in different cell types. In the small intestine, Stat3 is absolutely required for survival of the stem cells near the base of the crypt [7] and expression of dominant negative Stat3 in hematopoietic stem cells results in a reduced lympho-myeloid reconstituting ability [8]. In the mammary gland Stat3 is activated early during post-lactational regression and is a major regulator of the extensive cell death and tissue remodelling that occurs during this process [9], [10]. Recently, we demonstrated that activation of Stat3 is required during mammary gland involution to upregulate the expression of the lysosomal proteases, cathepsins B and L, and to downregulate the expression of their endogenous cytoplasmic inhibitor (Spi2A) thereby mediating cell death [11]. However, a potential role for Stat3 in mammary stem cells has not been determined.

Mammary epithelium consists of luminal (ductal and alveolar) and basal (myoepithelial) cells that are organised into a bi-layered structure with luminal cells lining the lumen encased by an outer layer of basal cells [12]. It is presumed that both luminal and basal lineages originate from common embryonic stem and progenitor cells. Moreover, each pregnancy cycle is accompanied by the massive expansion of the mammary epithelial compartment which suggests that the adult mammary gland contains a population of stem/progenitor cells with long-term self-renewal potential [13]. Previous reports have confirmed that mammary stem cells transplanted into a cleared fat pad can regenerate a functional mammary epithelial tree [14], [15], [16], [17]. Moreover, each full-term pregnancy cycle generates so called parity-induced mammary epithelial cells (PI-MECs) that produce milk proteins during late gestation and lactation and do not undergo programmed cell death during involution. Some of these cells act as alveolar progenitors during subsequent pregnancies and *in vivo* transplantation experiments proved their multipotency and self renewal capacity [18], [19]. Furthermore, these PI-MECs were shown to express cell surface markers that are associated with mammary stem cells [19].

Isolation of a pure mammary stem cell population has not been possible thus far due to lack of definitive markers. However, a mammary stem cell-enriched population can be obtained using a combination of cell surface markers and fluorescence-activated cell sorting (FACS). The population of CD24^+^ CD49f^hi^ cells contains basal cells, mammary stem cells and possibly luminal progenitors. Outgrowths arising from these cells are fully functional and able to produce milk when recipients are put through pregnancy [20], [21]. Moreover, mammary stem cells express basal markers such as keratin (K) 5, K14, smooth muscle actin (SMA), smooth muscle myosin, vimentin and laminin [20], [22]. Luminal cells are CD24^+^ CD49f^lo^, express K18 and lack expression of these basal markers. Luminal progenitors can be distinguished by the expression of the CD61 surface molecule and have the ability to form colonies *in vitro* in both two-dimensional and three-dimensional Matrigel culture [23].

Given the indispensable role of Stat3 in mESCs and intestinal crypt stem cells, and the essential role of Stat3 in mediating cell death during mammary gland involution, it was of interest to us to investigate the role of Stat3 in mammary gland-specific stem cells including both embryonic derived adult stem cells and those that are present following a full involution (PI-MECs).

## Materials and Methods

### Animal Husbandry

Mice bearing a *Stat3* gene flanked by loxP sites (*Stat3* ^fl/fl^) [24] were crossed with a strain expressing Cre recombinase gene under either the β-lactoglobulin (BLG) promoter [25] or the K14 promoter. K14-Cre mice were kindly donated by Dr. Michaela Frye (Centre for Stem Cell Research, Cambridge, UK), were originally from The Jackson Laboratories (Bar Harbor, ME, USA), and have a mixed C57Bl/6 × CBA background. All mice were maintained and bred in conventional cages within a specific pathogen free (SPF) animal facility. Immunodeficient CD1-Foxn1^−/−^ (nu/nu) nude mice were purchased from Charles River at the age of 22–28 days and maintained in individually ventilated cages (IVC) within a SPF animal facility. Animals were sacrificed through CO_2_ inhalation and/or dislocation of the neck. All animals were treated in strict accordance with the local ethical committee (University of Cambridge Licence Review Committee) and the UK Home Office guidelines. This study was specifically approved and authorised under the Project Licence of CJW.

### Genotype Analysis

In order to genotype *Stat3* ^fl/fl^ *;BLG-Cre* and *Stat3* ^fl/fl^ *;K14-Cre* mice and outgrowths, genomic DNA was isolated and the following primers were used in PCR reaction: BLG forward 5′-TCG TGC TTC TGA GCT CTG CAG-3′, BLG reverse 5′-GCT TCT GGG GTC TAC CAG GAA-3′, whey acidic protein (WAP) control forward 5′-CCT CCT CAG CAT AGA CA-3′, WAP control reverse 5′-GGT GAT CAG TCA CTT GCC TGA-3′, K14 forward 5′-TTC CTC AGG AGT GTC TTC GC-3′, K14 reverse 5′-GTC CAT GTC CTT CCT GAA GC-3′, K14 control forward 5′-CAA ATG TTG CTT GTC TGG TG-3′, K14 control reverse 5′-GTC AGT CGA GTG CAC AGT TT-3′, *Stat3* wt and floxed forward 5′-CAC CAA CAC ATG CTA TTT GTA GG-3′, *Stat3* wt and floxed reverse 5′-CCT GTC TCT GAC AGG CCA TC-3′, *Stat3* deleted forward 5′-CAC CAA CAC ATG CTA TTT GTA GG-3′, *Stat3* deleted reverse 5′-GCA GCA GAA TAC TCT ACA GCT C-3′.

### Semi-quantitative RT-PCR

RNA was extracted from sorted cells using TRIzol Reagent (Invitrogen) and cDNA was prepared using the Super Script First-Strand Synthesis System for RT (Invitrogen) following the manufacturer’s instructions. Semi-quantitative RT-PCR was performed with the following primers: Stat3, 5′-CAA TAC CAT TGA CCT GCC GAT-3′ and 5′-GAG CGA CTC AAA CTG CCC T-3′; Cyclophilin A, 5′-CCT TGG GCC GCG TCT CCT T-3′ and 5′-CAC CCT GGC ACA TGA ATC CTG-3′, and products were analysed on an agarose gel.

### Preparation of Single Cell Suspensions from Mammary Glands

Mammary tissues were collected from animals and digested at 37°C for 12–16 h in DMEM/F12 (Invitrogen) with 1% HEPES buffer (1 M, PAA) and 10 mg/ml collagenase (Roche) with 1000 U/ml hyaluronidase (Sigma). After the lysis of red blood cells in NH_4_Cl, cells were briefly digested with warm 0.25% Trypsin-EDTA, 5 mg/ml dispase (Sigma) and 1 mg/ml DNase (Sigma), and filtered through a 40 µm cell strainer (BD).

### FACS Analysis and Cell Sorting

Single cell suspensions were stained with biotinylated anti-CD31, anti-CD45 and anti-Ter119 antibodies, anti-CD24-PE (eBioscience), anti-CD49f-Alexa Flour 647, anti-CD61-Alexa Fluor 488 (BioLegend), streptavidin-PE Texas Red (BD) and propidium iodide (10 ng/ml; Sigma). Samples were filtered through a 30 µm cell strainer (Partec) immediately prior to flow cytometry analysis and sorting. Cells were either sorted using a MoFlo XDP sorter (DakoCytomation) or analysed using a CyAn™ ADP flow cytometer (DakoCytomation). The Summit 4.3 software (DakoCytomation) was used to analyse the data.

### Haematoxylin and Eosin (H&E) Staining and Immunohistochemistry

Haematoxylin and Eosin staining and immunohistochemistry were carried out as previously described [11], [26]. Primary antibodies used were: rabbit anti-phospho Stat5 (Cell Signalling Technology), mouse anti-E-cadherin (Cell Signalling Technology) and rabbit anti-Ki67 (Abcam). Secondary antibodies used were: Alexa Fluor 488 goat anti-mouse IgG (Invitrogen) and Cy3 goat anti-rabbit IgG (Invitrogen). Nuclei were stained with Hoechst 33342 (Sigma). The pictures were acquired using a Zeiss Axioplan 2 microscope.

### Whole Mounts

Mammary tissue was collected from female mice and stretched on a glass slide. Slides were incubated in Metha-Carnoy’s Fixative overnight and then stained with Carmine Alum overnight. After the Carmine had penetrated the entire tissue, the slides were placed in 100% ethanol for two hours and then in xylene for several hours. Samples were photographed using a Leica MZ7.5 stereomicroscope with a Leica DFC280 camera and Adobe Photoshop software.

### Colony Assay

NIH-3T3 fibroblasts were cultured in DMEM supplemented with 5% FCS and harvested from sub-confluent (<60%) cultures. Cells were irradiated by X-ray at 220 kV/14 mA for 14 min. Sorted mammary epithelial cells were collected in EpiCult-B Medium (Stem Cell Technologies) containing irradiated NIH-3T3 fibroblasts (10000 cells/ml), seeded on 6 cm polystyrene dishes (Nunc) and incubated at 37°C for one week. Then the colonies were fixed with ice cold methanol : acetone (1∶1) solution, stained with Giemsa and counted manually.

### Mammary Fat Pad Transplantation Assays

Basal cells were sorted and 25–1,000 cells were placed in 15 µl of HBSS (Invitrogen) supplemented with 1% FCS and 25% Matrigel™ Basement Membrane Matrix Growth Factor Reduced (BD). Mammary fat pad clearing and transplantation was performed on four-week-old CD1-Foxn1^−/−^(nu/nu) nude female mice (Charles River Lab). Mammary glands were collected 5 weeks after transplantation and processed as for whole mounts.

## Results and Discussion

**Figure 1:**
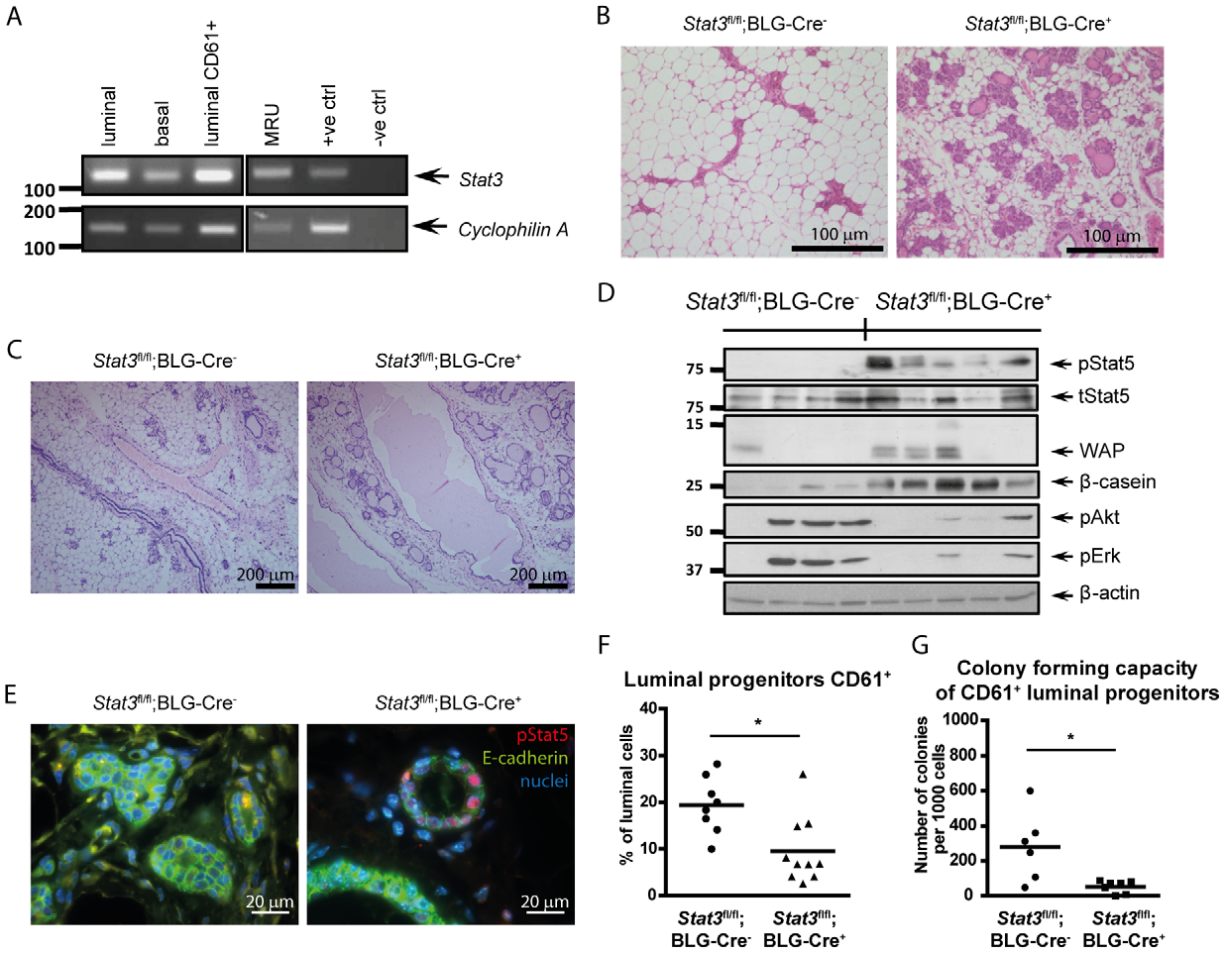
*Stat3^fl/fl^;BLG-Cre^+^* glands show incomplete involution and luminal progenitors have reduced proliferative capacity. (A) RT-PCR analysis of Stat3 expression in FACS sorted populations of mammary epithelial cells. MRU: mammary repopulating units. (B, C) H&E staining of sections of *Stat3^fl/fl^;BLG-Cre^−^* and *Stat3^fl/fl^;BLG-Cre^+^* mammary glands collected at day 5 of the second gestation (B) or four weeks after natural weaning (C). (D) Western blot analysis of four *Stat3^fl/fl^;BLG-Cre^−^* and five *Stat3^fl/fl^;BLG-Cre^+^* mammary glands four weeks after natural weaning for the expression or activation of Stat5, Erk, Akt, β-casein and WAP. β-actin was used as a loading control. (E) Immunohistochemistry staining for pStat5 (red) and E-cadherin (green) in mammary gland sections from *Stat3^fl/fl^;BLG-Cre^−^* and *Stat3^fl/fl^;BLG-Cre^+^* mice collected four weeks after natural weaning. Nuclei were stained with Hoechst 33342 (blue). (F) Flow cytometry analysis of luminal progenitors isolated from mammary glands of *Stat3^fl/fl^;BLG-Cre^−^* and *Stat3^fl/fl^;BLG-Cre^+^* females four weeks after natural weaning. (G) *In vitro* colony forming analysis performed on CD24^+^ CD49f^hi^ CD61^+^ luminal progenitor cells sorted from *Stat3^fl/fl^;BLG-Cre^−^* and *Stat3^fl/fl^;BLG-Cre^+^* mammary glands. Points represent the value for each mouse and lines depict mean values for each group. p value was determined using Student’s t test, * p<0.05.

In order to investigate the role of Stat3 in adult mammary gland stem cells, we determined initially if Stat3 is expressed in FACS sorted populations of mammary epithelial cells using RT-PCR. We detected Stat3 transcripts in all populations of cells tested including the mammary stem cell-enriched subpopulation of basal cells (mammary repopulating units, MRU), basal, luminal and luminal progenitor (CD61^+^) cells (Fig. 1A). As the β-lactoglobulin (BLG) promoter is activated primarily in the alveolar luminal epithelial cells of the mammary gland [27] and full recombination is achieved during lactation [25], we then used *Stat3^fl/fl^, BLG-Cre^+^* mice to conditionally delete *Stat3* in luminal mammary epithelium [11]. Since BLG-Cre and WAP-Cre drive recombination in the same populations of cells, deletion of Stat3 should occur also in PI-MECs following involution. In virgin animals, BLG is not widely expressed and drives recombination primarily in luminal ER^−^ progenitors, although recombination occurs in basal cells in both older (42-week-old) and parous (21-week-old) females [28]. In order to obtain maximum deletion of Stat3, *Stat3^fl/fl^;BLG-Cre^+^* females were taken through a pregnancy/lactation/involution cycle. Precocious development is evident during a second gestation in *Stat3^fl/fl^;BLG-Cre^+^* females with more alveolar structures and a reduced area occupied by adipocytes (Fig. 1B). This could reflect the retention of alveoli following involution or may be a consequence of effects downstream of Stat3 depletion on mammary stem and/or progenitor cells in terms of their number and functionality, thus resulting in alterations in the development of the gland during a second pregnancy. To discriminate between these possibilities we analysed mammary glands of *Stat3^fl/fl^;BLG-Cre^−^* and *Stat3^fl/fl^;BLG-Cre^+^* females after a “full involution” (four weeks after natural weaning). Strikingly, at this time point, glands with epithelial ablation of Stat3 showed incomplete involution with more intact alveolar structures and less adipose tissue compared to *Stat3^fl/fl^;BLG-Cre^−^* glands (Fig. 1C, Fig. S1). Moreover, we observed moderately to markedly ectatic ducts with normal cuboidal epithelium attenuated in the distended ducts (Fig. 1C). Analysis of protein levels revealed that glands from *Stat3^fl/fl^;BLG-Cre^+^* females have markedly increased levels of phospho-Stat5 (pStat5) and the milk proteins β-casein and whey acidic protein (WAP) (Fig. 1D, E). Normally, phosphorylation of Stat5 occurs during pregnancy and reaches the highest level in late gestation and early lactation [29]. This activation pattern is associated with an essential role for Stat5 in lobuloalveolar development [30], [31]. Furthermore, Stat5 was shown to be a survival factor during both involution and pregnancy [31], [32]. Thus, we speculate that the delayed involution observed in *Stat3^fl/fl^;BLG-Cre^+^* mice four weeks after natural weaning is partially a consequence of a pro-survival signal conveyed by activated Stat5, which also induces expression of milk proteins such as WAP and β-casein. However, Stat5 is required also for specification of early progenitors [33]. Therefore another possible interpretation is that deletion of Stat3 from basal MaSCs could result in precocious activation of Stat5, diminishing self-renewal potential and favouring specification of luminal progenitors.

Next we were interested in whether Stat3 deletion in mammary epithelium affects the relative numbers of different types of epithelial cells. To address this question, single-cell suspensions from *Stat3^fl/fl^;BLG-Cre^−^* and *Stat3^fl/fl^;BLG-Cre^+^* mammary glands four weeks after natural weaning were prepared, cells were stained for CD24, CD49f and CD61 antigens and analysed using flow cytometry [20], [23]. The following populations were distinguished within lineage negative (CD31*^−^* CD45*^−^* Ter119*^−^*) mammary cells in glands of both genotypes: CD24*^−^* CD49f^-^ stromal cells, CD24^+^ CD49f^lo^ luminal cells, and CD24^+^ CD49f^hi^ basal cells. Analysis of cell populations revealed that glands from *Stat3^fl/fl^;BLG-Cre^+^* mice did not show any difference in the number of luminal and basal cells (Fig. S2A, B). However, the population of CD24^+^ CD49f^lo^ CD61^+^ luminal progenitor cells was significantly reduced in *Stat3^fl/fl^;BLG-Cre^+^* females (Fig. 1F). CD61-positive luminal cells are luminal progenitors that have colony-forming capacity *in vitro* [23]. Thus we assessed the impact of *Stat3* deletion on the proliferative potential of luminal CD61^+^ progenitors in *in vitro* colony forming assays on a feeder layer of irradiated fibroblasts [23]. Surprisingly, CD61^+^ luminal progenitors isolated from *Stat3^fl/fl^;BLG-Cre^+^* glands four weeks after natural weaning showed significantly reduced capacity to form colonies compared to cells isolated from the *Stat3^fl/fl^;BLG-Cre^−^* glands (Fig. 1G). This may be a consequence of the reduced levels of pAkt and pErk that are present in cells deficient in Stat3 (Fig. 1D) and suggests that Stat3 plays a crucial role in maintenance of the proliferative capacity of CD24^+^ CD49f^lo^ CD61^+^ luminal progenitors. This result is all the more remarkable given that full deletion of both *Stat3* alleles was not obtained in cells from these *Stat3^fl/fl^;BLG-Cre^+^* mice (Fig. S3), suggesting biological selection against loss of the second *Stat3* allele.

**Figure 2:**
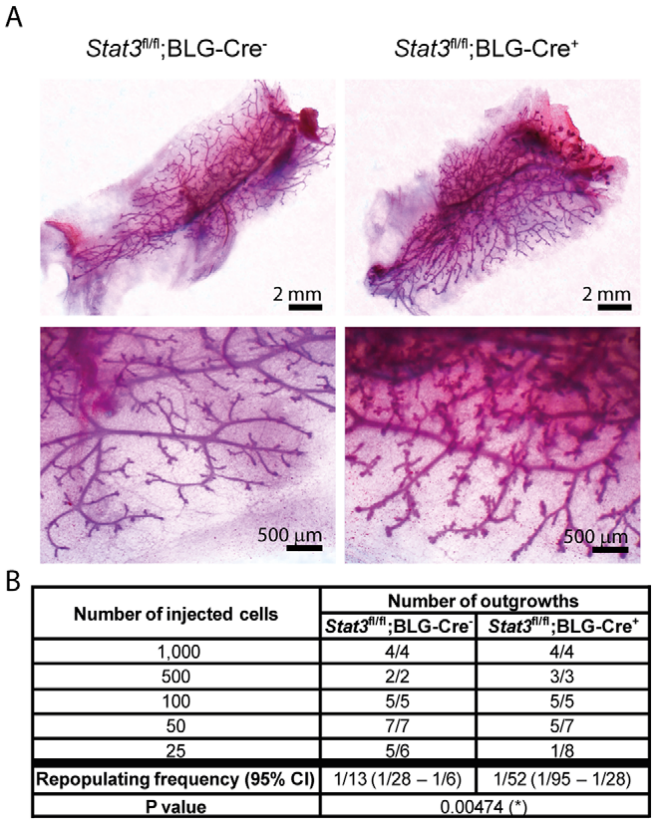
BLG-Cre mediated deletion of Stat3 affects repopulating frequency of stem cells and outgrowth phenotype. (A) Whole mount staining of mammary outgrowths originating from CD24^+^ CD49f^hi^ basal cells sorted from mammary glands of *Stat3^fl/fl^,BLG-Cre^−^* and *Stat3^fl/fl^;BLG-Cre^+^* females four weeks after natural weaning. (B) Limiting dilution analysis of the repopulating frequency of the mammary stem cell-enriched population sorted from mammary glands of *Stat3* ^fl/fl^,BLG-Cre*^−^* and *Stat3* ^fl/fl^;BLG-Cre^+^ females four weeks after natural weaning. Number of outgrowths is shown per number of transplanted fat pads. CI: confidence interval.

To assess the ability of mammary stem cells to repopulate the fat pad and form a normal mammary epithelial network in the absence of Stat3, the CD24^+^ CD49f^hi^ basal cells, which contain MaSCs, were sorted from glands of *Stat3^fl/fl^;BLG-Cre^−^* and *Stat3^fl/fl^;BLG-Cre^+^* females four weeks after natural weaning and cells were transplanted into cleared fat pads of immunocompromised nude mice. The outgrowths were analysed after five weeks. MaSCs isolated from both *Stat3^fl/fl^;BLG-Cre^−^* and *Stat3^fl/fl^;BLG-Cre^+^* mice were able to repopulate fat pads and generated ductal outgrowths with side branches (Fig. 2A). However, the outgrowths originating from *Stat3^fl/fl^;BLG-Cre^+^* cells were phenotypically different and displayed more side branching than those from *Stat3^fl/fl^;BLG-Cre^−^* cells. Limiting dilution transplantation assays were performed and analysed using Extreme Limiting Dilution Analysis software (http://bioinf.wehi.edu.au/software/elda/) [34]. These data showed that the repopulating frequency of the CD24^+^ CD49f^hi^ MaSC-enriched population from *Stat3* ^fl/fl^ *;BLG-Cre^+^* mice was four-fold lower than that of the *Stat3^fl/fl^;BLG-Cre^−^* cells after transplantation (Fig. 2B). In order to confirm that these outgrowths originated from mammary stem cells with long-term repopulating capacity, we performed secondary fat pad transplantation experiments. Primary outgrowths obtained from injection of 1,000 basal cells from *Stat3^fl/fl^;BLG-Cre^−^* or *Stat3^fl/fl^;BLG-Cre^+^* glands into cleared fat pads were collected and enzymatically dispersed. Subsequently, 20,000 cells from single cell suspensions were injected into the cleared fat pads of immunocompromised mice. After five weeks, the fat pads were checked for secondary outgrowths. We did not observe any differences in terms of the number of outgrowths or their size between *Stat3^fl/fl^;BLG-Cre^−^* and *Stat3^fl/fl^;BLG-Cre^+^* groups (Fig. S4). This suggests that, although there were fewer MaSCs in *Stat3* ^fl/fl^ *;BLG-Cre^+^* glands following involution, mammary stem cells from both *Stat3^fl/fl^;BLG-Cre^−^* and *Stat3^fl/fl^;BLG-Cre^+^* glands have a similar self-renewal potential.

**Figure 3:**
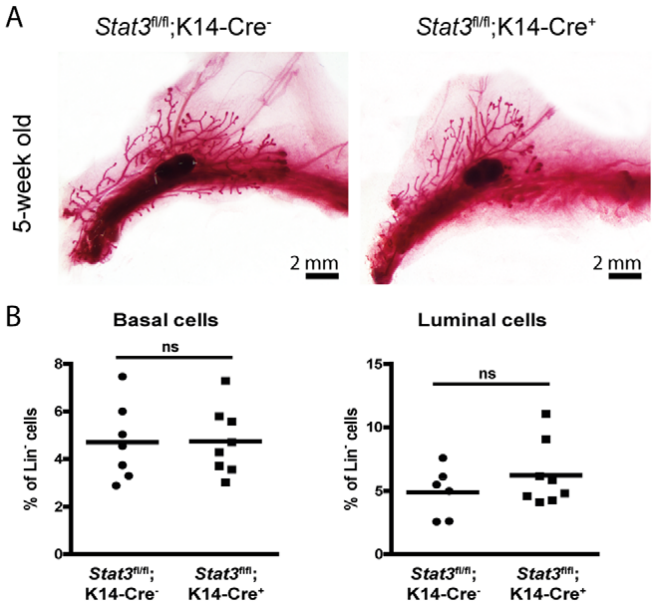
Normal mammary gland development in Stat3 depleted glands. (A) Whole mount staining of mammary glands of 5-week-old *Stat3^fl/fl^;K14-Cre^−^* and *Stat3^fl/fl^;K14-Cre^+^* females. (B) Flow cytometry analysis of luminal and basal cells isolated from mammary glands of *Stat3^fl/fl^;K14-Cre^−^* and *Stat3^fl/fl^;K14-Cre^+^* females four weeks after natural weaning. p value was determined using Student’s t test. ns: not significant.

**Figure 4:**
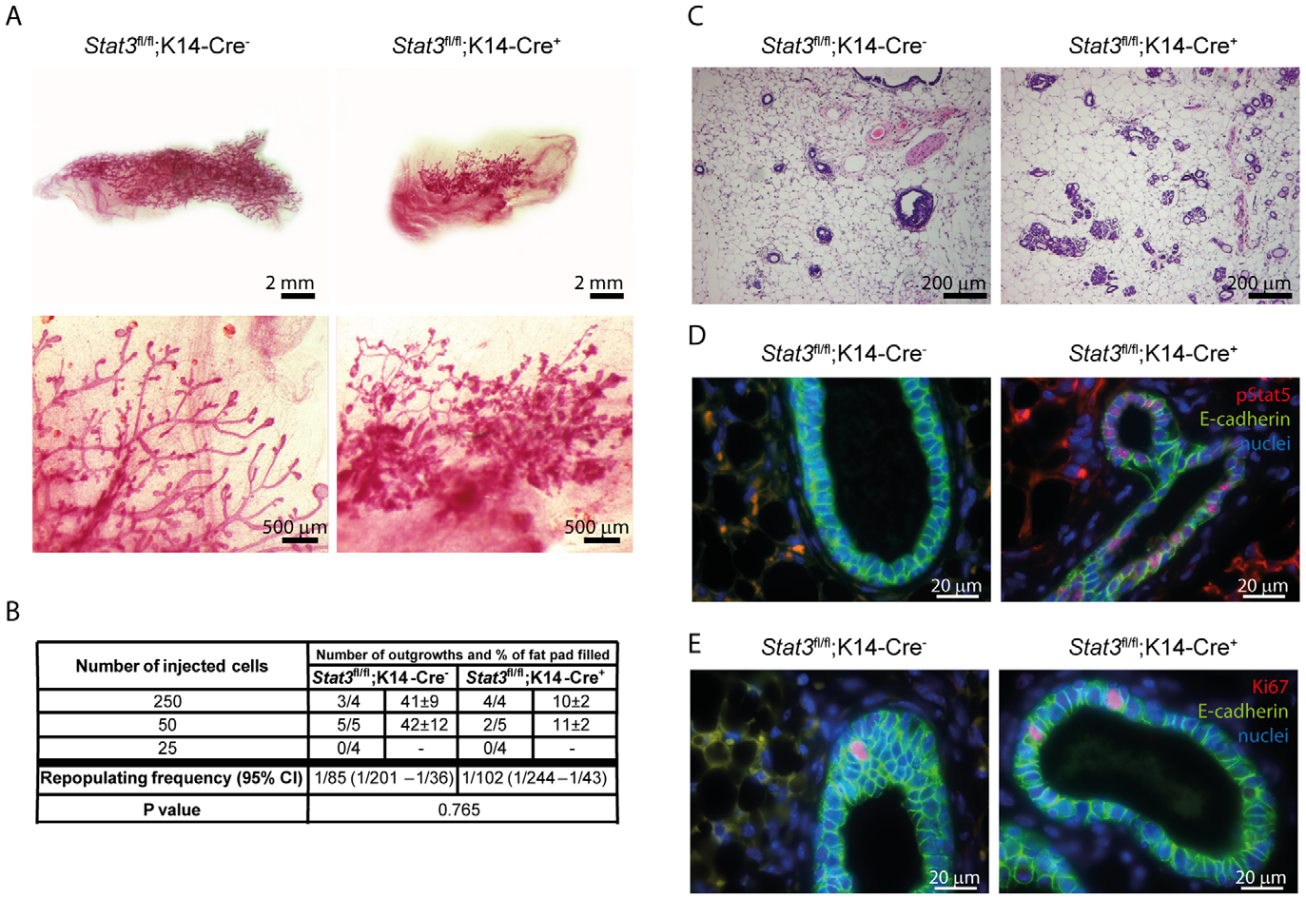
Stat3 is required to maintain the multipotency of mammary stem cells and their proliferative potential. (A) Whole mount staining of mammary outgrowths originating from CD24^+^ CD49f^hi^ basal cells sorted from mammary glands of 5-week-old *Stat3^fl/fl^,K14-Cre^−^* and *Stat3^fl/fl^;K14-Cre^+^* females. (B) Limiting dilution analysis to assess the repopulating frequency of the mammary stem cell-enriched population sorted from mammary glands of 5-week-old *Stat3^fl/fl^,K14-Cre^−^* and *Stat3^fl/fl^;K14-Cre^+^* females. Number of outgrowths per number of transplanted fat pads and percentage of fat pad filled ± standard error of the mean are shown. CI: confidence interval. (C) H&E staining of mammary outgrowths originating from CD24^+^ CD49f^hi^ basal cells sorted from mammary glands of 5-week-old *Stat3^fl/fl^;K14-Cre^−^* and *Stat3^fl/fl^;K14-Cre^+^* females. (D, E) Immunohistochemistry staining for pStat5 (red, D), Ki67 (red, E) and E-cadherin (green) in mammary outgrowths originating from CD24^+^ CD49f^hi^ cells from mammary glands of 5-week-old *Stat3^fl/fl^,K14-Cre^−^* and *Stat3^fl/fl^;K14-Cre^+^* females. Nuclei were stained with Hoechst 33342 (blue).

Interpretation of the fat pad transplantation data from parous *Stat3^fl/fl^;BLG-Cre* mice is confounded by the possibility that outgrowths originated either from MaSCs that had activated the BLG promoter and deleted the *Stat3* gene or from PI-MECs that have multipotent properties, can give rise to outgrowths upon transplantation, and express basal population markers [18], [19]. In order to further refine our investigation of a role for Stat3 in MaSCs so as to exclude PI-MECs we utilized a K14-Cre transgene crossed with *Stat3^fl/fl^* mice. This experimental setting allowed conditional Stat3 deletion in all K14 expressing cells in the embryo. Recently, Van Keymeulen and coworkers demonstrated that embryonic K14^+^ mammary stem/progenitor cells give rise to all mammary epithelial cell lineages [35]. *Stat3^fl/fl^;K14-Cre^+^* mice do not show any phenotypic changes compared to their *Stat3^fl/fl^;K14-Cre^−^* counterparts and pre-pubertal mammary gland development progresses normally regardless of *Stat3* deletion in K14-expressing cells (Fig. 3A, B). Moreover, *Stat3^fl/fl^;K14-Cre^+^* dams do not exhibit any lactation defects and can nurse pups normally (data not shown). This could be due to sufficient expression of Stat3 from the undeleted alleles (Fig. S5). However, transplantation of the CD24^+^ CD49f^hi^ basal cells sorted from glands of *Stat3^fl/fl^;K14-Cre^−^* and *Stat3^fl/fl^;K14-Cre^+^* females into cleared fat pads of immunocompromised nude mice revealed striking differences in the extent of fat pad filling with the Stat3 depleted cells giving rise to very small outgrowths that did not fill the fat pad regardless of the number of cells transplanted (Fig. 4A, B).This suggests a diminished ability of Stat3 depleted stem cells to proliferate. Secondly, the structure of the glands was different with normal ductal branching evident for the control transplants but a lack of long ducts coupled with disorganised highly branched lobular structures apparent in the *Stat3^fl/fl^;K14-Cre^+^* outgrowths in both whole mounts and H&E stained sections (Fig. 4A, C). These are similar to the outgrowths obtained from cells of the *Stat3^fl/fl^;BLG-Cre^+^* mice. This phenotype is reminiscent of that observed following transplantation of PI-MECs which frequently exhibit lobule-lineage restricted growth [36]. Moreover, this phenotype is apparent throughout the transplanted glands suggesting that reduction in the amount of Stat3 is sufficient to promote commitment to the alveolar lineage at the expense of the ductal lineage. This speculation is supported by analysis of nuclear pStat5 which is elevated in the outgrowths of *Stat3^fl/fl^;K14-Cre^+^* females compared to *Stat3^fl/fl^;K14-Cre^−^* females (Fig. 4D) as observed also for the fully involuted *Stat3^fl/fl^;BLG-Cre^+^* glands. However, levels of proliferation were not significantly different in *Stat3^fl/fl^;K14-Cre^+^* and *Stat3^fl/fl^;K14-Cre^−^* outgrowths (Fig. 4E). These data indicate that the multipotent capacity of basal cells, which is lost following birth, cannot be re-acquired when Stat3 is depleted suggesting that Stat3 could be required for reprogramming adult mammary stem cells to their multipotent state. *In vitro* culture of basal cells isolated from *Stat3^fl/fl^;K14-Cre^−^* virgin glands in 3D Matrigel organoid culture [37] gave rise to branched solid organoids as expected while basal cells from *Stat3^fl/fl^;K14-Cre^+^* glands produced rounded hollow organoids, similar to those formed by luminal cells (data not shown). In the light of these data, we suggest that Stat3 is also important for the maintenance of luminal progenitor proliferative potential.

## Supporting Information

Figure S1 **Incomplete involution of mammary glands with BLG-Cre mediated epithelial ablation of Stat3.** Whole mount staining of mammary glands of *Stat3^fl/fl^;BLG-Cre^−^* and *Stat3^fl/fl^;BLG-Cre^+^* females, collected four weeks after natural weaning.
(TIF)

Figure S2 **BLG-Cre mediated epithelial ablation of Stat3 does not affect the number of luminal and basal cells.** Flow cytometry analysis of luminal (A) and basal (B) cells isolated from mammary glands of *Stat3^fl/fl^;BLG-Cre^−^* and *Stat3^fl/fl^;BLG-Cre^+^* females four weeks after natural weaning. Points represent the value for each mouse and lines depict mean values for each group. p value was determined using Student’s t test. ns: not significant.
(TIF)

Figure S3 **Analysis of** ***Stat3*** **alleles in mammary gland cell populations from** ***Stat3^fl/fl^;BLG-Cre*** **mice.** (A) Representative gel showing *Stat3* floxed and deleted alleles in genomic DNA isolated from unsorted and sorted basal, luminal, stromal and lineage positive (Lin^+^) cells from mammary glands of *Stat3^fl/fl^;BLG-Cre^−^* (C) and *Stat3^fl/fl^;BLG-Cre^+^* (KO) females four weeks after natural weaning. Different amounts of genomic DNA were used for each PCR reaction. (B) Quantification of the *Stat3* deleted to floxed alleles ratio in unsorted and sorted basal and luminal cells from panel A.
(TIF)

Figure S4 **Mammary stem cells from** ***Stat3^fl/fl^;BLG-Cre*** *^−^* **and** ***Stat3^fl/fl^;BLG-Cre^+^*** **mice have similar long-term repopulating capacity.** (A) Whole mount staining of secondary outgrowths obtained after injection of 20,000 cells from the mammary glands arising from primary outgrowths of *Stat3^fl/fl^;BLG-Cre^−^* and *Stat3^fl/fl^;BLG-Cre^+^* cells transplanted into cleared fat pads. (B) Number of secondary outgrowths per number of transplanted fat pads.
(TIF)

Figure S5 **Analysis of** ***Stat3*** **alleles in skin and mammary gland cell populations from** ***Stat3^fl/fl^;K14-Cre*** **mice.** (A, B) Representative gels showing *Stat3* floxed and deleted alleles in genomic DNA isolated from skin (A), mammary gland tissue and sorted basal and luminal mammary cells (B) from 5-week-old *Stat3^fl/fl^;K14-Cre^−^* (C) and *Stat3^fl/fl^;K14-Cre^+^* (KO) females. Different amounts of genomic DNA were used for each PCR reaction. (C) Quantification of the *Stat3* deleted to floxed alleles ratio in skin, mammary tissue and sorted basal and luminal cells from panels A and B.
(TIF)
