# Supplementary figures and images for: Stat3 Is Required to Maintain the Full Differentiation Potential of Mammary Stem Cells and the Proliferative Potential of Mammary Luminal Progenitors
Source: PLoS One. 2012 Dec 20;7(12):e52608. doi: 10.1371/journal.pone.0052608 (PMC3527594; doi:10.1371/journal.pone.0052608)

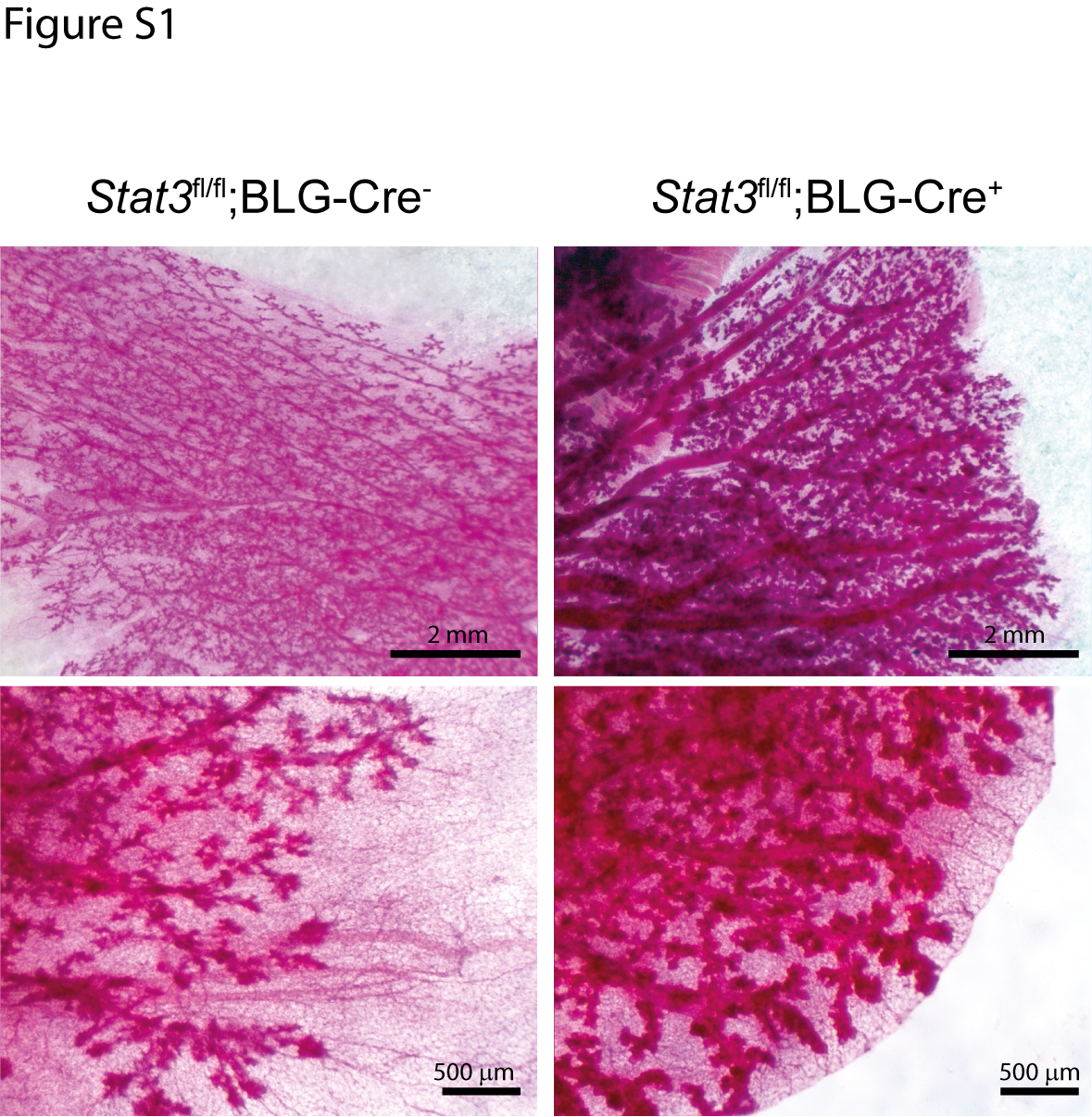

Supplement: Figure S1 — Incomplete involution of mammary glands with BLG-Cre mediated epithelial ablation of Stat3. Whole mount staining of mammary glands of Stat3fl/fl;BLG-Cre− and Stat3fl/fl;BLG-Cre+ females, collected four weeks after natural weaning. (TIF) [file pone.0052608.s001.tif]

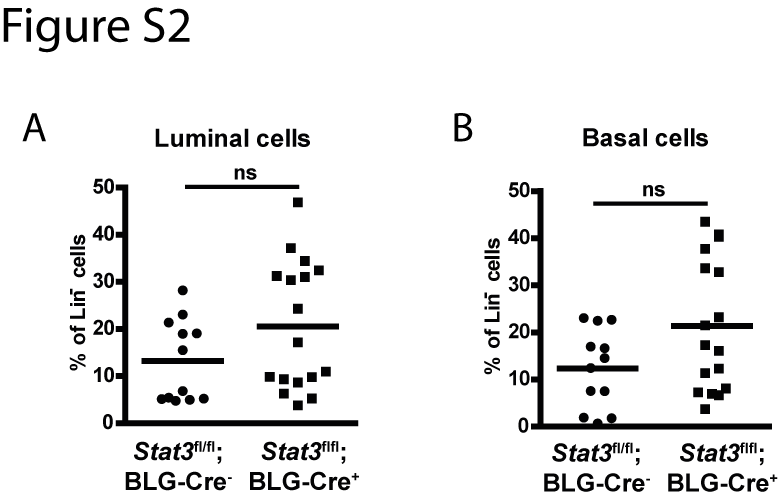

Supplement: Figure S2 — BLG-Cre mediated epithelial ablation of Stat3 does not affect the number of luminal and basal cells. Flow cytometry analysis of luminal (A) and basal (B) cells isolated from mammary glands of Stat3fl/fl;BLG-Cre− and Stat3fl/fl;BLG-Cre+ females four weeks after natural weaning. Points represent the value for each mouse and lines depict mean values for each group. p value was determined using Student’s t test. ns: not significant. (TIF) [file pone.0052608.s002.tif]

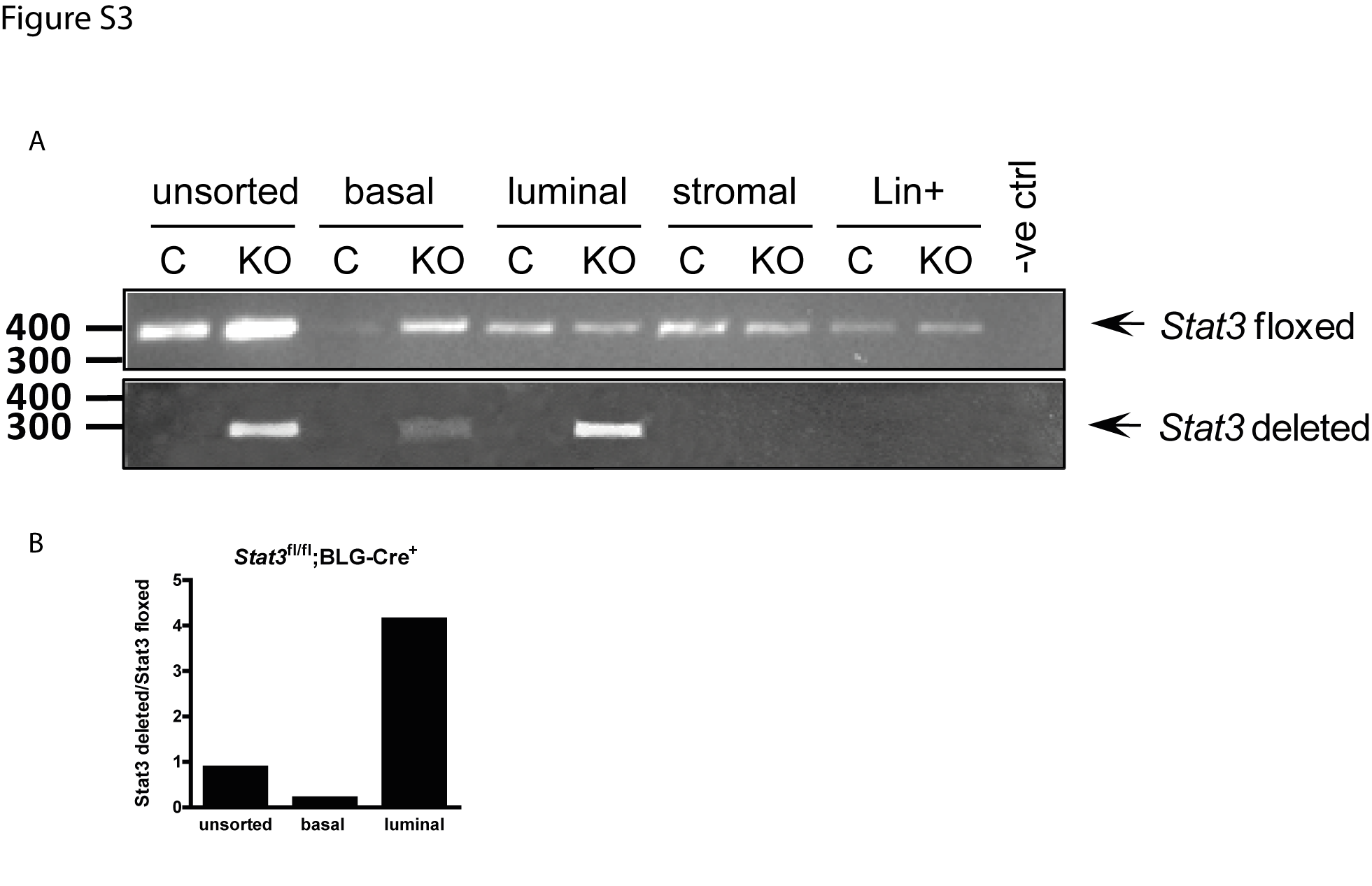

Supplement: Figure S3 — Analysis of Stat3 alleles in mammary gland cell populations from Stat3fl/fl;BLG-Cre mice. (A) Representative gel showing Stat3 floxed and deleted alleles in genomic DNA isolated from unsorted and sorted basal, luminal, stromal and lineage positive (Lin+) cells from mammary glands of Stat3fl/fl;BLG-Cre− (C) and Stat3fl/fl;BLG-Cre+ (KO) females four weeks after natural weaning. Different amounts of genomic DNA were used for each PCR reaction. (B) Quantification of the Stat3 deleted to floxed alleles ratio in unsorted and sorted basal and luminal cells from panel A. (TIF) [file pone.0052608.s003.tif]

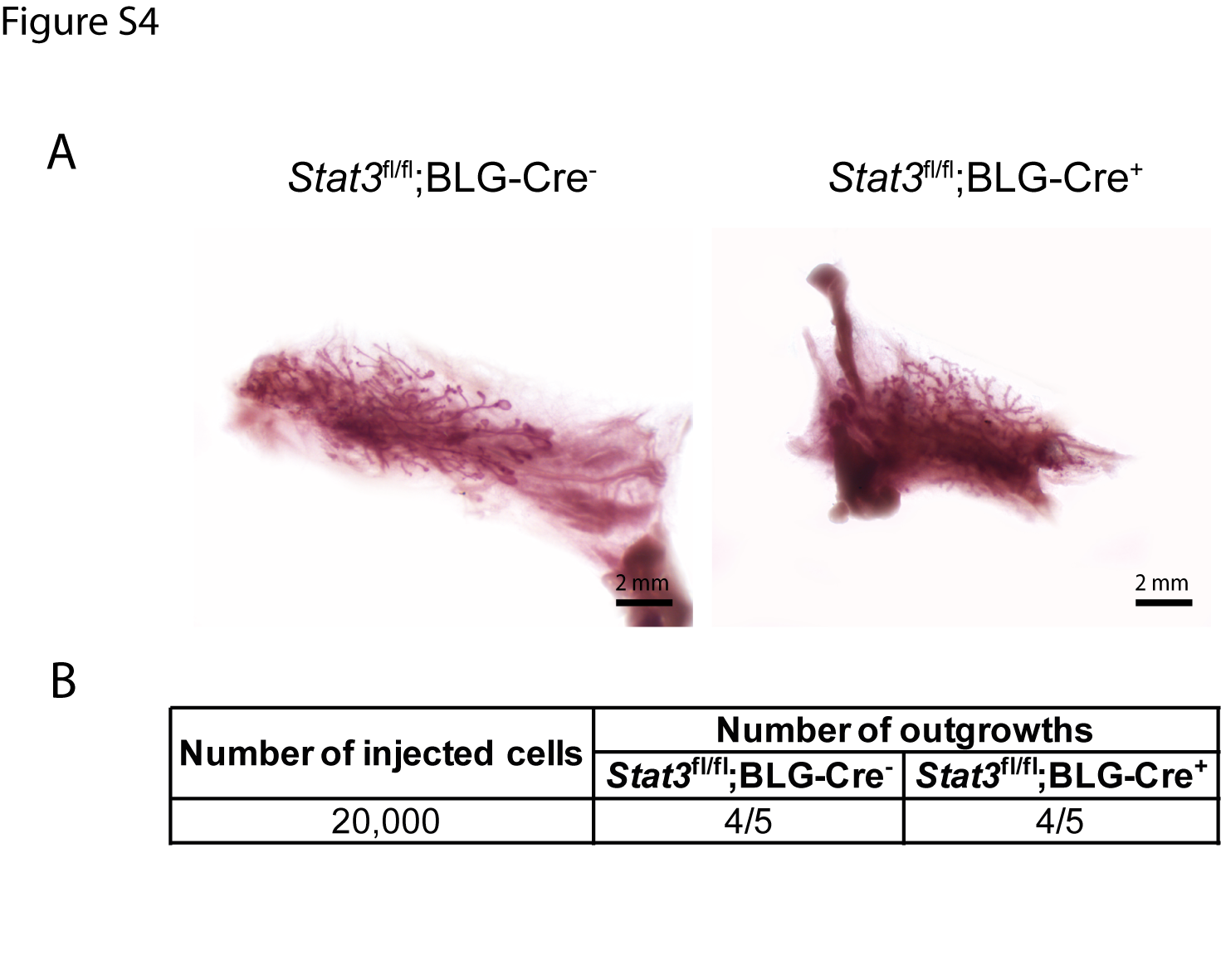

Supplement: Figure S4 — Mammary stem cells from Stat3fl/fl;BLG-Cre − and Stat3fl/fl;BLG-Cre+ mice have similar long-term repopulating capacity. (A) Whole mount staining of secondary outgrowths obtained after injection of 20,000 cells from the mammary glands arising from primary outgrowths of Stat3fl/fl;BLG-Cre− and Stat3fl/fl;BLG-Cre+ cells transplanted into cleared fat pads. (B) Number of secondary outgrowths per number of transplanted fat pads. (TIF) [file pone.0052608.s004.tif]

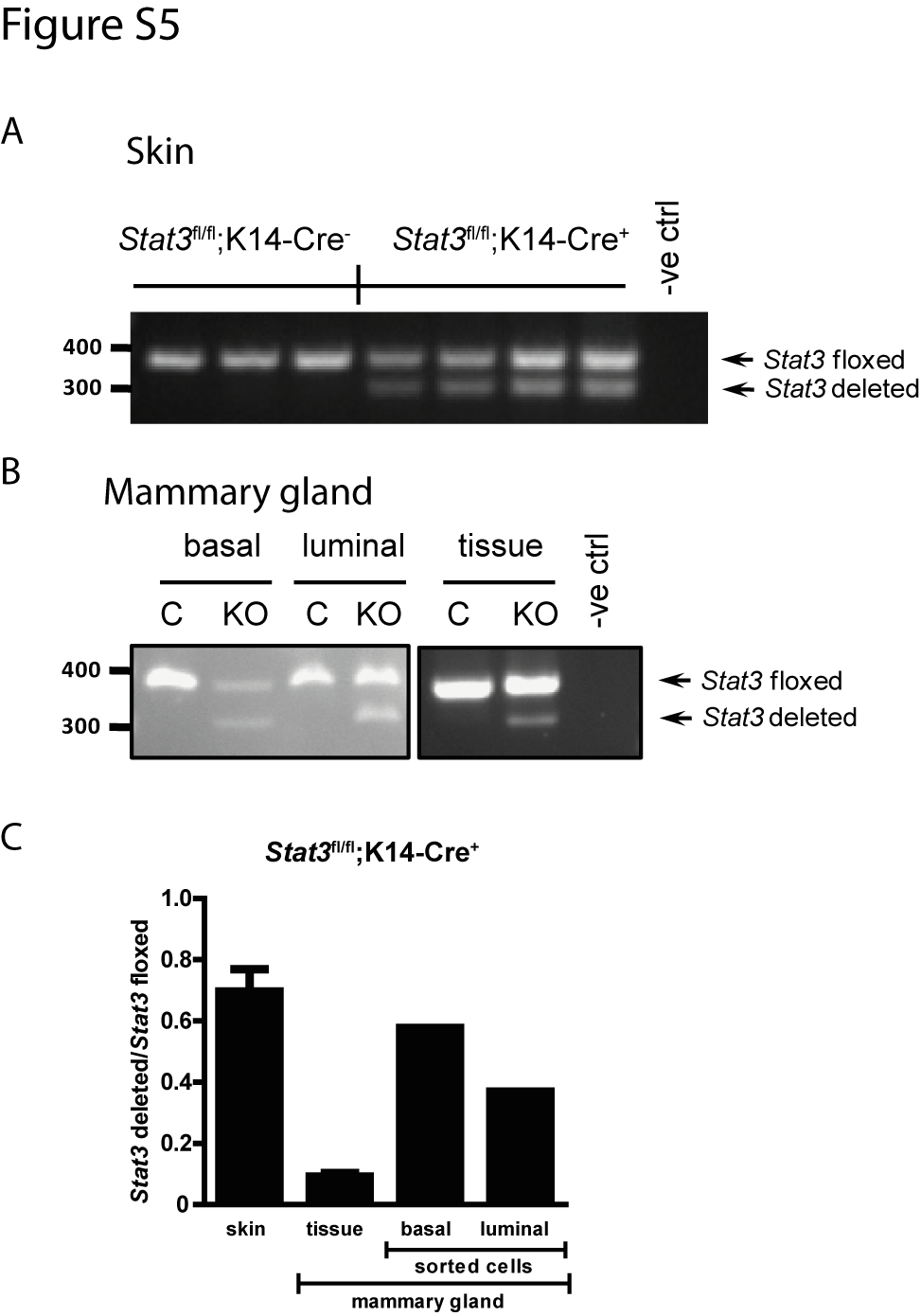

Supplement: Figure S5 — Analysis of Stat3 alleles in skin and mammary gland cell populations from Stat3fl/fl;K14-Cre mice. (A, B) Representative gels showing Stat3 floxed and deleted alleles in genomic DNA isolated from skin (A), mammary gland tissue and sorted basal and luminal mammary cells (B) from 5-week-old Stat3fl/fl;K14-Cre− (C) and Stat3fl/fl;K14-Cre+ (KO) females. Different amounts of genomic DNA were used for each PCR reaction. (C) Quantification of the Stat3 deleted to floxed alleles ratio in skin, mammary tissue and sorted basal and luminal cells from panels A and B. (TIF) [file pone.0052608.s005.tif]
